# Radiation Exposure and Mortality from Cardiovascular Disease and Cancer in Early NASA Astronauts

**DOI:** 10.1038/s41598-018-25467-9

**Published:** 2018-05-31

**Authors:** S. Robin Elgart, Mark P. Little, Lori J. Chappell, Cato M. Milder, Mark R. Shavers, Janice L. Huff, Zarana S. Patel

**Affiliations:** 1 University of Houston, Houston, Texas USA; 2 Radiation Epidemiology Branch, National Cancer Institute, DHHS, NIH, Division of Cancer Epidemiology and Genetics, Bethesda, Maryland USA; 3 KBRwyle, Science and Space Operations, Houston, Texas USA; 4 NASA Lyndon B. Johnson Space Center, Houston, Texas USA; 5 MEI Technologies, Houston, Texas USA

**Keywords:** Cancer, Cardiovascular diseases, Epidemiology

## Abstract

Understanding space radiation health effects is critical due to potential increased morbidity and mortality following spaceflight. We evaluated whether there is evidence for excess cardiovascular disease or cancer mortality in early NASA astronauts and if a correlation exists between space radiation exposure and mortality. Astronauts selected from 1959–1969 were included and followed until death or February 2017, with 39 of 73 individuals still alive at that time. Calculated standardized mortality rates for tested outcomes were significantly below U.S. white male population rates, including all-cardiovascular disease (n = 7, SMR = 33; 95% CI, 14–65) and all-cancer (n = 7, SMR = 43; 95% CI, 18–83), as anticipated in a healthy worker population. Space radiation doses for cohort members ranged from 0–78 mGy. No significant associations between space radiation dose and mortality were found using logistic regression with an internal reference group, adjusting for medical radiation. Statistical power of the logistic regression was <6%, remaining <12% even when expected risk level or observed deaths were assumed to be 10 times higher than currently reported. While no excess radiation-associated cardiovascular or cancer mortality risk was observed, findings must be tempered by the statistical limitations of this cohort; notwithstanding, this small unique cohort provides a foundation for assessment of astronaut health.

## Introduction

Since the beginning of the space program, NASA has recognized the potential risk of adverse health effects due to the stressors associated with manned spaceflight^1^. These stressors include altered gravity, isolation, a closed environment, and distance from Earth. The omnipresent space radiation environment is of particular concern because the associated health effects pose increased morbidity and mortality risks that may impact mission operations and limit mission duration. Potential health effects include those threatening mission success and those impacting long-term health, including the focus of the current study: cardiovascular disease (CVD) and cancer^2,3^.

Although risks in low Earth orbit (LEO) are not thought to be substantial^4^, as NASA prepares for extended missions beyond LEO, health risks associated with space radiation exposure become a larger concern. Outside Earth’s protective magnetosphere, astronauts are exposed to higher levels of galactic cosmic rays (GCR) that have physical characteristics which are distinct from terrestrial sources of radiation such as x-rays and gamma-rays. GCR consists of high atomic number and high energy (HZE) nuclei (1%) as well as high energy protons (90%) and helium particles (9%) present at chronic and low dose-rates^5^. HZE ions produce densely ionizing tracks and impart clustered DNA damage difficult for cells to repair. Also, delta rays emanating from the particle track can impact neighboring cells, contributing further damage^6,7^. These distinctive properties of space radiation complicate extrapolation of risk estimation because the human biological effects of the spectrum of particles present in space radiation are not well-characterized compared to those of terrestrial radiation.

There is considerable epidemiological evidence from large-scale cohort studies linking low dose, low dose-rate, and low-LET terrestrial radiation exposures to development of leukemias and solid cancers^8–11^. There is also an accumulating body of evidence showing an association between moderate and low doses of radiation (<0.5 Gy) and increased risk of CVD after an estimated latency of 10 years or more^12–15^. However, possible effect modification by well-known lifestyle factors for CVD (e.g. smoking and obesity) obscure interpretation of findings in the low dose range, making it more difficult to detect significant differences from background disease without a large study population^16^.

A recent report claims astronauts may have a higher than anticipated risk of CVD associated with space radiation exposure^17,18^ but that finding has been disputed, largely due to shortcomings in the statistical assumptions and methodologies used in the study^19,20^. While other published reports compare deaths in the astronaut corps to the general population^21,22^, to our knowledge, only one analysis to date has evaluated the relationship between individual astronaut radiation exposure histories and mortality. This previous analysis, however, used estimated space radiation doses and only considered deaths from cancer and “all natural causes”^23^. Here, we perform a thorough epidemiological investigation of the early NASA astronaut cohort followed until death or February 16, 2017 (whichever occurred first), to determine if evidence exists of excess risk of cancer and CVD, specifically ischemic heart disease (IHD), cerebrovascular disease (CeVD), and all-CVD, and to determine if a correlation exists between space radiation exposure and disease mortality^24^. Small cohort size and low radiation exposure levels likely limit the statistical power of studies investigating this cohort. Therefore, a critical part of the current analysis is an assessment of analytical power.

## Results

### Population description

This early astronaut cohort is composed of 73 white males, born between 1921 and 1942 and selected as astronauts between 1959 and 1969 (NASA selection groups 1–7), contributing a total of 3,120.8 person-years. These astronauts were followed until death or February 16, 2017 (whichever occurred first); their follow-up covers the time frame of interest, that is, the time frame in which radiation effects are seen in adults post-exposure. Furthermore, there is a gap in NASA astronaut selection between 1969 and 1978 when selection criteria evolved which provides a convenient cutoff for cohort inclusion to improve cohort homogeneity. Individuals participated in the Mercury, Gemini, Apollo, Skylab, Apollo-Soyuz, and Shuttle programs, and their time in space ranged from 0–3 months. Mean current age of those still alive at study end was 83.4 years (SD = 3.4). Cause of death by major category (cancer, CVD, accident, other, unknown), and other descriptive statistics are presented by space radiation dose category (Table 1).

**Table 1:** Early astronaut cohort demographics binned by total space radiation dose category.

Total Space Radiation Dose (mGy)	<0.2	0.2–1.99	2–3.99	4–10.99	≥11	Total
# Astronauts	14	19	11	15	14	73
# Cancer Deaths	2	2	1	0	2	7
# Cardiovascular Disease Deaths	1	4	1	1	0	7
# Accident Deaths	6	5	0	0	1	12
# Other Deaths	1	0	1	0	1	3
# Unknown Deaths	1	0	0	3	1	5
Mean Medical Dose (SD)	2.4 (6.4)	27.7 (13.6)	34.4 (20.8)	29.1 (15.6)	32.5 (21.7)	25.1 (19.4)
Mean Year at Birth (SD)	1932.6 (4.1)	1931.7 (5.2)	1931.6 (2.5)	1932.2 (4.4)	1931.5 (3.3)	1931.9 (4.1)
Mean Age at Entry into Astronaut Corps (SD)	31.6 (2.7)	32.2 (3.4)	33.0 (2.5)	31.8 (2.8)	32.5 (2.2)	32.2 (2.8)
Mean Follow up Time (SD)	29.3 (23.6)	40.3 (15.0)	46.4 (12.9)	50.7 (7.8)	48.1 (7.5)	42.8 (16.1)
Total Group Person Years	409.9	766.5	510.1	760.8	673.4	3120.8
Mean Age at Death (SD)	57.7 (23.8)	65.7 (15.9)	64.5 (14.9)	78.2 (19.9)	74.9 (10.2)	65.2 (19.1)
Mean Current Age of Living Astronauts (SD)	79.9 (2.9)	82.1 (3.9)	84.9 (3.1)	83.6 (3.6)	83.8 (2.3)	83.4 (3.4)

SD = standard deviation.

### Radiation dosimetry

**Figure 1:**
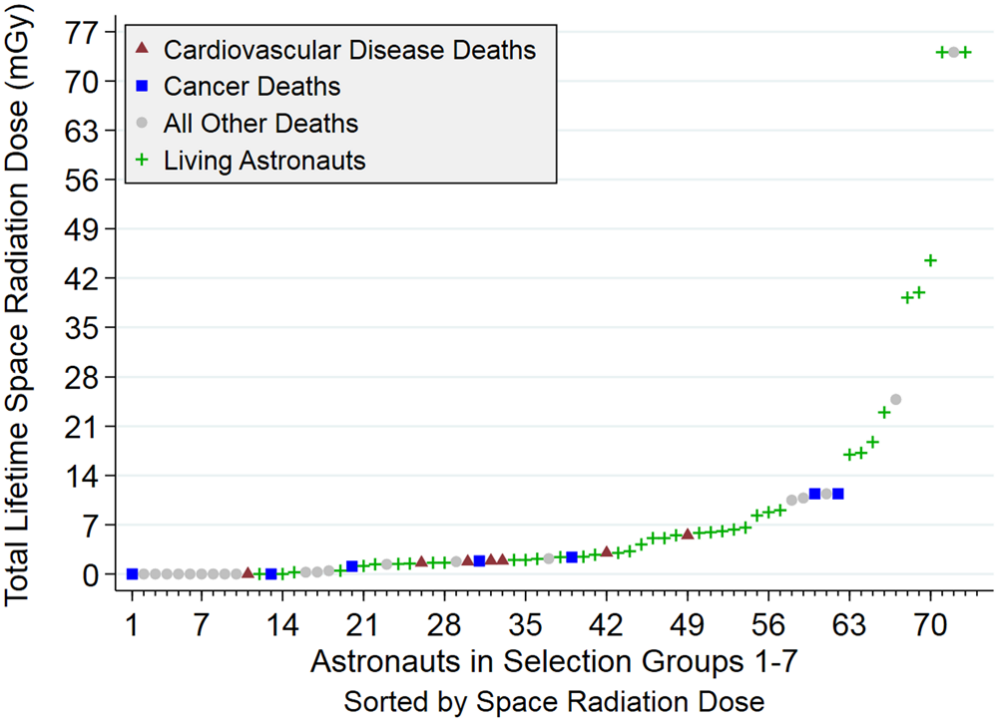
Individual Astronaut Dosimetry. Summary of individual dosimetry and vital status for all NASA crew in astronaut selection groups 1–7. Dosimetry, presented as total mean skin dose in mGy, was obtained from published records; median dose was 2 mGy with a range of 0–74.1 mGy. Living astronauts are indicated by (**+**); while deaths are distinguished by major cause into three groups, cardiovascular disease (▲), cancer (■) or other (•).

Astronaut occupational radiation doses include both space and medical exposures. Mean medical radiation effective dose was 25.1 mGy (SD = 19.4). Space radiation doses vary widely depending on mission parameters such as time in the solar cycle, mission duration and trajectory^25^. Figure 1 provides a summary of individual space radiation doses, with associated vital status. Total lifetime space radiation absorbed doses ranged from 0–74.1 mGy (median = 2; SD = 16.3). While the composition of the radiation environment is unique for each mission, with the exception of the outer Van Allen electron belt (which is effectively shielded by the vehicle and stowage), protons are the major flux component for low earth orbit and free space missions and are expected to contribute the majority of absorbed dose. GCR contains the highest percentage of helium and heavier, more highly charged particles, which account for approximately 10% of the baryon flux impinging on vehicles that are outside the Earth’s magnetosphere. The other natural sources of intra-vehicular space radiation exposure (inner Van Allen belt and solar particle events) are composed almost entirely of protons^5^. The ionization rate for a charged particle determines the absorbed dose and is approximately proportional to Z^2^, where Z is the particle’s charge number^26^. Thus, for a free-space GCR-only radiation environment with a composition of 90% protons, 9% helium, and 1% heavy ions, approximately 50% of the dose contribution is due to protons, 20% due to helium, and 30% due to heavier ions assuming an average heavy ion charge of 8. Given that GCR is only a fraction of the total radiation dose for a specific mission, and nuclear interactions in shielding create a multiplicity of protons, these values reflect an overestimation of the dose contribution for particles heavier than protons. Gamma rays are at most a trivial contributor to space radiation dose. A thorough analysis using detailed radiation transport and dose modeling is required to accurately assess the contributions to organ dose from each particle species for each astronaut’s mission. Although this type of analysis was not conducted for the current report, previous studies have assessed the space environment in this way^27–30^.

Individual lifetime space radiation doses were all <7 mGy for CVD deaths and <14 mGy for cancer deaths. Of the astronauts who received >14 mGy, 1 died of other causes, 1 died of unknown causes, and 9 were still alive at study end.

### Survival and mortality

As of February 16, 2017, of the 73 cohort members, 34 (47%) have died (Table 1). Accidents (n = 12, 16%; 8 NASA-related and 4 non-NASA-related) were the leading cause of death, followed by cancer (n = 7, 10%), and CVD (n = 7, 10%). SMRs were calculated by specific causes using age- and calendar year-matched U.S. white males as the comparison population (Table 2)^31^. Observed values represent recorded deaths in the astronaut cohort while expected values represent the number of deaths expected in an age- and calendar year-matched U.S. white male comparison population of 73 individuals. SMR values <100 signify a lower mortality rate by specified cause in the astronaut cohort compared to the U.S. white male population, while values >100 indicate higher mortality rates. Calculated SMRs and 95% confidence intervals for cancer (SMR = 43; 95% CI, 18–83), all-CVD (SMR = 33; 95% CI, 14–65), and IHD (SMR = 40; 95% CI, 13–89) were all significantly lower compared to the U.S. white male population. Only for CeVD did the upper confidence interval for the SMR include 100, indicating it was not significantly different from the comparison population (SMR = 77; 95% CI, 9–268) (Table 2). The SMR for accidents was significantly elevated above 100 (SMR = 536; 95% CI, 287–1913) (Table 2), as expected in a population with high-risk occupations.

**Table 2:** Observed vs. expected major causes of death, standardized mortality ratios.

Endpoint	ICD8 range	ICD9 range	ICD10 range	Observed	Expected	SMR (95% CI)
All-cardiovascular disease	390–459	390–459	I00–I99	7	21.1	33 (14, 65)
Ischemic heart disease	410–414	410–414	I20–I25	5	12.5	40 (13, 89)
Cerebrovascular disease	430–438	430–438	I60–I69	2	2.6	77 (9, 268)
All-cancer (benign and malignant)	140–239	140–239	C00–C99, D00–D48	7	16.5	43 (18, 83)
Accidental mortality	E800–E929	E800–E869, E880–E928	V01–X59	12	2.2	536 (287, 1913)
All-cause mortality	0-796, E800-E999	001-799, E800-E999	A00-Y89	34	58.0	59 (44, 74)

The potential association of space radiation exposure with disease mortality in this astronaut cohort was investigated using a logistic regression model; the lower dose astronauts served as comparisons for the higher dose astronauts using a continuous dose variable. Table 3 shows trends of risk, given as the natural log of the odds ratio (OR) per Gy (ln[OR]/Gy) of space radiation dose for five outcomes (all-CVD, IHD, CeVD, cancer, all-cause mortality). For all outcomes considered, trends with lifetime space radiation dose were negative, suggesting decreased risk with increasing radiation dose. Trends remained negative regardless of whether there was adjustment for age at exit from the cohort, age at both entrance into the cohort and exit, medical radiation dose, or year of birth. However, none of these results were statistically significant at the α < 0.05 level. The lack of effect of adjustment for medical dose suggests that it does not confound the space radiation dose-response.

**Table 3:** Radiation risk estimates (excess ln[Odds Ratio] per Gy) for four major causes of death in relation to absorbed dose*.

	ln[OR]/Gy (95% CI)				
	All-cardiovascular disease (ischemic heart and cerebrovascular disease)	Ischemic heart disease	Cerebrovascular disease	Cancer	All-cause mortality
Number of deaths	7	5	2	7	34
Absorbed dose adjusted for age at exit	−116.4 (−462.1, 16.1)	−60.0 (−382.9, 34.3)	−580.7 (−600.3^a^, 22.9)	−43.0 (−224.3, 24.2)	−8.2 (−52.8, 24.6)
*p*-value	0.14	0.37	0.10	0.25	0.70
Absorbed dose adjusted for age at exit and entrance	−120.1 (−474.0, 16.1)	−60.7 (−389.3, 34.6)	−616.1 (−636.8^a^, >0^a^)	−42.1 (−230.3, 25.1)	−11.4 (−58.7, 26.0)
*p*-value	0.14	0.37	0.10	0.32	0.56
Absorbed dose adjusted for age at exit and entrance, medical diagnostic dose^b^	−123.5 (−491.6, 16.9)	−62.7 (−411.8, 32.6)	−501.5 (−925.1^a^, 32.9)	−46.3 (−230.7, 26.9)	−4.6 (−56.2, 35.6)
*p*-value	0.14	0.35	0.11	0.30	0.84
Absorbed dose adjusted for age at exit and entrance, year of birth, medical diagnostic dose^b^	−124.4 (−496.7, 16.9)	−68.8 (−429.6, 35.8)	−385.5^c^ (−407.6^a^, −363.5^a^)	−46.7 (−265.9, 36.6)	−0.5 (−70.3^a^, 69.3^a^)
*p*-value	0.14	0.35	0.25^c^	0.39	0.57

*Unless otherwise indicated, all confidence intervals are profile-likelihood based.

^a^Wald-based CI.

^b^Categorical variable 0–14 mGy, 15–29 mGy, >30 mGy.

^c^Indications of non-convergence.

### Power analysis

Results of the power analysis for logistic regression over the outcomes IHD, CeVD, all-CVD, and all-cancer mortality are presented in Tables 4 and 5. Statistical power for all outcomes was <6% when previously reported excess relative risks (ERRs) per Gy were assumed (Table 4)^14,32^. Power estimates remained under 12% even when assuming (1) 10 times the reported ERR/Gy, and (2) 10 times the currently observed number of deaths. Table 5 presents an additional analysis performed to assess the number of deaths or risk level required for each cause of death to achieve 80% statistical power. If risks are assumed to be 10 times higher than those recorded in the literature, ≥2,025 cancer deaths and ≥4,479 CVD deaths would need to be observed to achieve 80% power. Likewise, ERRs/Gy would have to be at least 50 times greater than those currently observed to reach 80% power, even when 10 times the current number of deaths is assumed.

**Table 4:** Power using a 1-sided test of trend (with type I error α = 0.05)*.

Endpoint	Deaths	EOR^a^/Gy	Power (%)*
Cerebrovascular disease (CeVD)	**2**	**0**.**308**	**5**.**1**
	**2**	3.08	5.3
	20	**0**.**308**	5.2
	20	3.08	7.0
Ischemic heart disease (IHD)	**5**	**0**.**147**	**5**.**0**
	**5**	1.47	5.4
	50	**0**.**147**	5.1
	50	1.47	6.5
All-cardiovascular disease (using CeVD EOR/Gy)	**7**	**0**.**308**	**5**.**1**
	**7**	3.08	6.1
	70	**0**.**308**	5.3
	70	3.08	9.1
All-cardiovascular disease (using IHD EOR/Gy)	**7**	**0**.**147**	**5**.**1**
	**7**	1.47	5.5
	70	**0**.**147**	5.2
	70	1.47	6.8
All malignant cancer	**7**	**0**.**47**	**5**.**2**
	**7**	4.70	6.7
	70	**0**.**47**	5.5
	70	4.70	11.8

*Power is evaluated using the asymptotic method of Little *et al*.^56^.

^a^Excess Odds Ratio.

Bolded numbers represent values observed in the present study, expected EORs/Gy from literature, and associated power. Remaining numbers represent 10x values observed in the present study or 10x expected EORs/Gy from literature, and associated power.

**Table 5:** Analysis of predicted number of deaths and risk levels required to achieve 80% power in logistic regression assuming the observed dose distribution*.

Endpoint	Deaths	EOR^a^/Gy
Cerebrovascular disease (CeVD)	**2**	>10^6^
	20	101.52
	4479	3.08
	407,689	**0**.**308**
Ischemic heart disease (IHD)	**5**	654.31
	50	46.44
	18,635	1.47
	1,779,569	**0**.**147**
All-cardiovascular disease (using CeVD EOR/Gy)	**7**	353.08
	70	36.12
	4479	3.08
	407,689	**0**.**308**
All-cardiovascular disease (using IHD EOR/Gy)	**7**	353.08
	70	36.12
	18,635	1.47
	1,779,569	**0**.**147**
All malignant cancer	**7**	353.08
	70	36.12
	2025	4.70
	176,084	**0**.**47**

*Power is evaluated using the asymptotic method of Little *et al*.^56^.

^a^Excess Odds Ratio.

Bolded numbers represent values observed in the present study or expected EORs/Gy from literature.

## Discussion

The radiation environment in space is a major concern for long duration missions due to the potential for human health effects. Elevated cancer risk is a well-established late outcome following exposure to ionizing radiation at the dose levels anticipated during exploration missions. Recent terrestrial epidemiology studies indicate lower exposure thresholds for cardiovascular effects that approach heart exposures predicted for a Mars exploration mission^13^, elevating the concern for cardiovascular disease from low dose radiation exposure. As NASA extends missions further from Earth and for longer durations, radiation exposures will increase and health risks are likely to become more of a concern.

To investigate mortality rates in this early astronaut cohort compared to a U.S. white male population, SMRs were calculated for six mortality outcomes. SMRs indicate a lower risk of CVD, cancer, and overall mortality in early NASA astronauts. Twelve of the 34 deaths in this population were due to accidents, which impacted lifespan considerably. As shown in Table 2, despite an elevated SMR for all accidents, the SMR for all deaths, a measure that includes accidents, is significantly less than that of the U.S. white male population. This result is expected given the overall healthy status of astronauts, who undergo rigorous selection and training. Furthermore, it is consistent with previous analyses showing increased longevity and reduced SMRs for cancer and CVD in astronauts compared to the U.S. average population^21,33^. These results demonstrate a healthy worker effect in this cohort.

As expected in a healthy worker population, excess risk of CVD or cancer mortality has never been observed in U.S. astronauts when appropriate methodologies are used for comparison to other populations, such as the general U.S. population^20–22^ or the NASA Johnson Space Center civil service employees^34–36^. A previous study describing a higher CVD mortality ratio among Apollo lunar astronauts reported only the proportional mortality ratio^17^. This methodology is inappropriate to the circumstances of the cohort, as the specific assumptions and conditions required to use the proportional mortality ratio to compare mortality across groups are not met^19,20,37^. A better approach is to use age-standardized data to compare mortality to that of the U.S. white male population; however, the paucity of deaths does not lend itself to direct standardization. The reported SMRs generated from indirect standardization have provided some insight, but the small number of deaths remains an obstacle in any analysis of the NASA astronaut cohort^38^. However, even this approach is not optimal. The U.S. white male population has serious limitations as a comparison group for this cohort because astronauts are subject to extraordinary and repeated selection processes and would be expected to be much fitter than the general population, as indeed we have demonstrated (Table 2).

The best approach to assessing the effect of radiation (and other) exposures on astronaut health is an internal analysis, as for example, the logistic regression conducted here. This study is the first to employ this type of regression analysis to assess the effect of space radiation exposure on mortality in an astronaut cohort. The results do not support an association between radiation and any studied outcome, including both CVD and cancer. The lack of any significant correlations is not surprising given the small cohort size and low radiation doses, resulting in low statistical power. Therefore, this work also assessed the power of the logistic regression to detect changes in radiation-associated risks. The current data include 7 deaths from CVD and 7 deaths from cancer. With this limited number of events, statistical power is <6% for all endpoints. Power remains <12% even when risks are assumed to be 10 times those reported in the literature^19^ and when the number of deaths is estimated at 10 times those recorded. Over 400,000 deaths from CVD and over 170,000 deaths from cancer would need to be observed to achieve 80% power at currently documented risk levels (Table 5). This analysis indicates the limited number of non-accidental deaths in this cohort is insufficient to attribute any of these deaths to radiation exposure. The power analysis conducted here can be contrasted with the generic analysis of power conducted by the United Nations Scientific Committee on the Effects of Atomic Radiation (UNSCEAR), which used an artificial population with dose distribution modeled on the Japanese atomic bomb survivors to determine power to detect a linear trend in dose as a function of the assumed ERR/Gy. For ERR/Gy values under 0.5, the implied numbers of deaths (i.e. cases) required for 80% power were on the order of at least 1000^24^.

The small number of astronauts, the small number of deaths, and the resulting low power in the logistic regression analysis are limitations in this study. With only 73 astronauts and 34 deaths, it is unlikely that statistical significance will be observed unless radiation risks are well above those currently reported in the literature. Furthermore, the high number of astronauts dying in accidents reinforces the effect of low power, since these individuals must be either excluded or controlled for in analyses of radiation risks. It is standard practice to adjust for the role of time on mortality in regression analyses. When age and year of birth are adjusted together, one is equivalently adjusting for calendar year. In the logistic regression in this study, adjusting for year of birth or age at cohort entry and exit did not change the results. Due to these results and low power, further adjustments for other time-related parameters such as age at first exposure and latency time were not considered.

An additional limitation in studying the early astronaut population is the inability to test for confounding or effect modification by health and lifestyle variables (e.g. smoking and drinking) due to a lack of information both from astronauts and the U.S. white male population. While future studies should also attempt to test for effect modification by lifestyle factors, it is unlikely that there are any confounding effects of strong carcinogens, such as cigarette smoking, as these are often limited by the small correlations between radiation exposure and the putative confounding carcinogen. As shown by Blair *et al*., the confounding of associations between lung cancer risk and a large number of other occupational carcinogens by cigarette smoking is generally modest^39^. There is also little evidence that smoking and other major lifestyle/medical risk factors for circulatory disease appreciably modify the radiation risk in those cohorts where this information is available^14^.

Dosimetric uncertainty is a limitation of many radio-epidemiological studies, including the present research. Similar to studies of nuclear workers^40^, errors in astronaut dosimetry result from uncertainties in the position of the film badge on the subject and the relative orientation of the subject in relation to the radiation field. This uncertainty is compounded by the inability to control for non-NASA radiation exposures. In principle, adjustment can be made for the effects of such errors^41^. However, we lack the information on the magnitude of errors necessary to undertake a corrected analysis. Unlike nuclear worker cohorts, doses are available for each astronaut, spanning their total time in the space radiation environment. As such, uncertainties in dosimetry are unlikely to be much larger than in the nuclear worker studies^40^, and could be smaller. Additionally, limited understanding of radiation quality necessitates the use of absorbed skin doses (mGy) rather than the more biologically meaningful effective or equivalent doses (mSv). The latter are scaled to specific disease outcomes based on relative biological effectiveness (RBE) values. It is expected that different types of particle radiations will have RBE values different from one, regardless of disease type, but the precise values are not well understood for space radiations. Furthermore, certain mission parameters, perhaps most importantly the amount of physical shielding, affect the specific mixture of particle radiations experienced by an astronaut, and these parameters are not generally known^4,42,43^.

Finally, as discussed earlier, the healthy worker effect exists in this cohort in that the astronauts are a highly selected group of healthy individuals, notwithstanding the high accident mortality rate. This limitation is demonstrated in the SMR analysis where astronauts show low or similar mortality compared to the U.S. population in every outcome except accident mortality.

There are also several key strengths in this study. One strength is the use of an internal reference for the logistic regression analysis. Due to the healthy worker effect, an appropriate reference population is key in designing a study of astronaut disease incidence and mortality. The use of the internal reference nearly eliminates the healthy worker effect.

Another strength stems from controlling for medical exposures in the astronaut cohort to more accurately assess the risk from space radiation exposure. Although no effect was seen after controlling for the medical doses, it is good practice to ensure that the lack of an association does not result from exposures otherwise unaccounted for. Variations in the organs exposed, the number of diagnostic exams received, and the type of diagnostic exams received (e.g. projection x-ray, dual exposure x-ray absorptiometry, computerized tomography) are expected due to the unique medical needs of individual astronauts, as well as the time period in which the exams were performed. The majority of exams were diagnostic projection x-rays, as documented in Peterson *et al*.^21^ and in the Longitudinal Study of Astronaut Health summary report^44^. Although we do not consider exam type, this should not affect the current analysis, as we are only concerned with total medical dose for adjustment purposes, as noted above.

While the criterion of 80% power is typically used to establish initial sample sizes for biomedical studies, this work demonstrates how problematic this convention is when analyzing health effects in small populations. To achieve 80% power in a logistic regression for this cohort, the number of observed deaths would need to be two orders of magnitude higher than currently recorded, even if radiation risks are assumed to be 10 times higher than observed terrestrially. In practice, significant effects can still be observed regardless of whether a study achieves 80% power, but as power decreases these significant effects are likely to be increasingly biased^45^. Continued monitoring is the only means available to provide the necessary epidemiological assessment of spaceflight exposures even though the epidemiological data is likely to remain limited and caution should be used when interpreting results from astronaut-based cohort studies. Despite the recognized difficulties in direct analysis of spaceflight risks in astronauts, researchers at NASA must extract meaningful data from the limited number of humans who have the opportunity to live and work in space each year^46^. Considering the unique challenges in assessing this cohort, the most promising approach for future radiation research in astronaut populations would be a longitudinal study with a suitable control population (for instance, test pilots or astronauts with minimal deep space exposure), recording cases as they occur. The astronaut cohort is currently monitored over time as part of the Lifetime Surveillance of Astronaut Health (LSAH), a NASA sponsored occupational surveillance program for astronauts^47^, so this type of study is feasible given an appropriate control population can be identified.

In order to supplement and validate conclusions from longitudinal follow-up of astronauts, the current research strategy combining low dose human epidemiology data with data from cellular and animal models will be continued. Within the NASA Human Research Program, the Space Radiation Element focuses on advancing the space radiobiology state-of-knowledge through animal and cellular model systems and space radiation exposures simulated at the NASA Space Radiation Laboratory. This approach provides essential evidence for appropriate translation of health risks from terrestrial radiation epidemiology to space radiation risk assessment and development of mitigation strategies to protect the crew both during and after flight.

In summary, standardized mortality ratios show a reduced risk of all-cause mortality, cancer, and all-cardiovascular disease in early NASA astronauts compared to the U.S. white male population, as expected in a highly selected, healthy worker population. Furthermore, no excess radiation-associated risk of cancer or cardiovascular disease mortality was observed in logistic regression with an internal reference group. All findings must be tempered by the statistical limitations of this small cohort, since unique characteristics of the astronaut cohort limit the power to detect an association between radiation exposure and mortality. A research strategy combining low dose human epidemiology data with cellular and animal results from heavy particle exposures can be utilized for space radiation risk assessment in astronaut cohorts.

## Methods

### Outcome measures

The primary outcomes assessed in this analysis were CVD and cancer, where CVD subtypes CeVD and IHD were also individually analyzed. Additionally, all-cause mortality was considered.

### Data collection

NASA astronauts from selection groups 1–7, hired between 1959 and 1969, were selected for inclusion in this analysis. This relatively homogeneous cohort contains exclusively white males with published space radiation doses and complete follow-up over the time frame in which radiation effects are seen in adults post-exposure^19,48,49^. All astronauts in this cohort were followed until time of death or February 16, 2017, whichever occurred first. Cause of death (identified on death certificates) and demographic information were obtained from the LSAH program at Johnson Space Center (Table 1). The International Classification of Diseases, 10^th^ revision (ICD-10), was used to classify cause of death from death certificates. ICD-10 codes were matched to corresponding ICD-8 and ICD-9 codes to link cause of death in the astronauts to that of the general population for each year of study. Table 2 details specific ICD-8, -9, and -10 codes used.

### Radiation dosimetry

Space radiation exposures were compiled from previously published mission specific doses from thermoluminescent dosimeter measurements^19,48,49^. These doses represent averages from all personal crew dosimeters aboard a specific mission. Mission-averaged doses were summed as appropriate for each astronaut and are presented as lifetime total space radiation dose categories (Table 1). Space radiation doses are presented as absorbed skin doses (mGy) rather than equivalent or effective doses (mSv), since equivalent and effective doses are scaled specifically to carcinogenic effects and are not appropriate for analysis of other outcomes (e.g. CVD). Total occupational medical effective radiation doses were collected from astronaut radiation histories, de-identified, and categorized for privacy protection prior to analysis. It was judged that because relative medical doses are comparable in magnitude with space radiation doses and possibly could be correlated, doses from medical procedures could potentially confound the space-radiation dose response and should be adjusted for in the analysis. Additionally, because medical radiation (unlike space radiation) irradiates the body in a highly non-uniform way and is expected to carry an RBE equal to 1, it was determined that effective dose was a better measure to use for medical exposures.

### Statistical analysis

Disease mortality rates of this early astronaut cohort were calendar year- and age-matched to those of the U.S. white male population, using Centers for Disease Control (CDC) WONDER data for single calendar years and standard decades of age (15–24, 25–34, 35–44, …, 75–84, ≥85 years of age)^31^. CDC Wonder data was available from 1968-present; for the years 1959–1967, the data from 1968 was applied. Standardized mortality ratios (SMRs) were calculated for the early astronaut population using these matched disease mortality rates. Exact binomial confidence intervals on each SMR were constructed^50^.

Since the healthy worker effect is likely present in this cohort, a model was fitted using an internal reference group. Preliminary attempts to fit a Cox proportional hazards model proved problematic, with convergence issues. A logistic regression model was therefore fitted to five principal mortality outcomes: (a) all-CVD (*n* = 7); (b) IHD (*n* = 5); (c) CeVD (*n* = 2); (d) all-cancer (*n* = 7); and (e) all-cause mortality (*n* = 34). A model was fitted in which the natural log odds ratio (ln[OR]) for probability (*p*) of the specific disease in individual *i* with lifetime space radiation dose *D*_*i*_ is given by:

1 $$ln[\frac{{p}_{i}}{1-{p}_{i}}]={\alpha }_{0}+\sum _{j}\,{\alpha }_{j}{Z}_{ij}+\beta {D}_{i}$$

for some adjusting variables *Z*_*ij*_ with dose as a continuous variable. The model parameters, *a*_*j*_, *β* were estimated by maximum-likelihood^51^, using Epicure^52^. The likelihood ratio test was used to assess whether addition of the dose trend parameter *β* to the baseline model significantly improved model fit^53^. Confidence intervals were constructed from the profile likelihood^51^, or when this failed, via Wald test inversion^54^. Particular emphasis is placed on two types of regression adjustment: one adjusting for age at exit from the cohort (death or end of study period) and the other adjusting for age at both entrance into the cohort (selection year) and exit, which is equivalent to adjusting for duration of follow-up and age at exit. Additional analyses adjusted for medical radiation dose, treated as a factor variable with levels 0–14, 15–29, and ≥30 mGy and year of birth. The analysis was performed fully blinded to the identities of the subjects by the principal statistician (M.P.L.).

### Analysis of statistical power

Statistical power of the logistic regression was estimated separately for IHD, CeVD, all-CVD, and all-cancer mortality using risk factors (ERR/Gy) taken from the literature^13,55^. In all cases, the power of a 1-sided test of trend with type I error rate (α) of 5% was determined using the methodology of Little *et al*.^56^. Both the observed number of deaths in this cohort and 10 times the number of observed deaths were tested for each outcome. The assumed ERR/Gy, which approximates the EOR/Gy, was 0.147 for IHD, 0.308 for CeVD, and both of these ERR/Gy assumptions were tested for all-CVD. These estimates were derived from a recent meta-analysis that included low dose-rate exposures^13^. An EOR/Gy of 0.47 was assumed for all-cancer deaths, derived from the estimated ERR/Gy for all solid cancer mortality in the Japanese atomic bomb survivors^55^. Power was also estimated assuming each of the above ERRs/Gy was multiplied by 10. Furthermore, calculations were performed to estimate number of deaths or risk level that would be required to achieve 80% power in a logistic regression analysis. No power assessment was made for all-cause mortality since there is no expected ERR/Gy associated with this outcome available in the literature applicable to astronauts.

### Data Availability

The datasets generated during and/or analyzed during the current study are available from the corresponding author upon request.

### Ethical considerations

A record of deceased U.S. NASA astronauts through February 16, 2017 was obtained from NASA Johnson Space Center (JSC) Lifetime Surveillance of Astronaut Health and NASA website (http://www.jsc.nasa.gov/Bios/astrobio_former.html). Because data were collected only on deceased astronauts or from publicly available records, informed consent and IRB approval was not required.
